# Evaluating Good Husbandry Practices and Organic Fermented Additives for Coccidiosis Control in a Pilot Study Using Slow-Growing Broilers

**DOI:** 10.3390/ani15121752

**Published:** 2025-06-13

**Authors:** Anabel E. Rodriguez, Jesica D. Britez, María Luz Pisón-Martínez, Fernando O. Delgado, Facundo Balbiani, Cecilia C. Berardo, César Gramaglia, Facundo Cuba, Tomás J. Poklepovich, Claudia Moreno, Gladys Francinelli, Gabriel Morici, Martín Arias, Javier Schapiro, Pablo Barbano, Mariela L. Tomazic

**Affiliations:** 1 Instituto de Patobiología Veterinaria (IPVET), Unidad de Doble Dependencia, Instituto Nacional de Tecnología Agropecuaria (INTA), Consejo Nacional de Investigaciones Científicas y Técnicas (CONICET), Hurlingham B1686, Argentina; 2 Instituto de Patobiología (IP), INTA, Hurlingham B1686, Argentina; 3 Cátedra de Parasitología y Enfermedades Parasitarias, Facultad de Ciencias Veterinarias, Universidad de Buenos (UBA), Ciudad Autónoma de Buenos Aires (CABA) C1427, Argentina; 4 Estación Experimental Agropecuaria (EEA), INTA, Luján B6700, Argentina; 5 EEA, INTA, Villa Dolores, Córdoba X5870, Argentina; gramaglia.cesar@inta.gob.ar; 6 Unidad Operativa Centro Nacional de Genómica y Bioinformática-ANLIS, Dr. Carlos G. Malbrán, Ciudad Autónoma de Buenos Aires (CABA) B1282, Argentina; 7 Cátedra Física, Facultad de Farmacia y Bioquímica (FFyB), Universidad de Buenos (UBA), Ciudad Autónoma de Buenos Aires (CABA) C1113, Argentina; 8 Cátedras de Parasitología y de Enfermedades Parasitarias, Facultad de Ciencias Agrarias y Veterinarias, Universidad del Salvador, AHU, Pilar B1630, Argentina; 9 Cátedra de Biotecnología, FFyB, Universidad de Buenos (UBA), Ciudad Autónoma de Buenos Aires (CABA) C1113, Argentina

**Keywords:** family farming poultry systems, Campero-INTA, coccidiosis, *Eimeria*, animal welfare practices, agroecology, lactic acid bacteria, productivity

## Abstract

The Argentine Campero-INTA slow-growing chicken, a widely used breed in family poultry farming, faces high coccidiosis prevalence, impairing productivity. Control often relies on management and drugs due to vaccination costs. This pilot study assessed the breed’s susceptibility to local *Eimeria* and the impact of good animal welfare practices (AWPs) and an organic fermented additive, locally produced, combined with AWPs (OF-AWPs). Two trials evaluated productive (body weight gain and feed conversion), infection (oocyst excretion and lesion score), and histopathological parameters (villus height and crypt depth). The productivity (PI) and anticoccidial (ACI) indexes were calculated. Metagenomic analysis of the additive was also conducted. Mild to moderate coccidiosis significantly reduced PI (7.99–16.83 vs. 29.29 in unchallenged controls). In the second trial, AWPs showed good anticoccidial efficacy (ACI 173.9), while OF-AWPs demonstrated high efficacy, especially in birds of 28 days (ACI 180.6), improving productive parameters, reducing oocyst shedding, and enhancing the villus height to crypt depth ratio. Over a 75-day cycle, the OF-AWP increased the PI by 24.44% compared to untreated chickens (108.8 vs. 87.43). Lactic acid bacteria were the main component of the organic fermented additive. This research highlights the potential of an agroecological strategy to manage coccidiosis in Campero-INTA chickens.

## Simple Summary

To ensure healthy and sustainable food systems, it is crucial to adopt a ‘One Health’ approach, which recognizes the interconnectedness of human, animal, and environmental health. In this context, family poultry farms in Argentina play an important role in providing high-quality meat and eggs, contributing significantly to food security. However, these farms often face challenges, particularly from coccidiosis, a widespread parasitic disease that severely impacts chicken health, reduces productivity, and causes economic losses. While large-scale poultry operations use vaccinations, family farms typically rely on management practices and medications to control coccidiosis due to cost constraints. This pilot study aimed to assess the susceptibility of a chicken breed commonly used by small Argentine producers to local *Eimeria* spp. and to explore sustainable coccidiosis management. The results showed that even mild *Eimeria* infection, primarily of moderate pathogenicity, decreased productive parameters. However, combining good husbandry practices, including animal welfare, with an organic fermented additive improved these parameters. These findings suggest that environmentally friendly approaches could effectively control this prevalent parasitic disease, reducing the need for anticoccidial chemicals, offering significant economic benefits to small-scale farmers, and potentially extending their applicability to other slow-growing breeds worldwide.

## 1. Introduction

Globally, poultry production is continuously expanding: protein availability from poultry is projected to grow 17.8% by 2030, and by then, poultry meat is expected to represent 41% of all protein sources [1]. Sustainable systems that embrace the One Health approach are essential. Family farming is key for food security in low- and middle-income countries, providing high-quality food and diversified products, predominantly involving rural women and offering opportunities for economic independence [2]. In Argentina, family farming represents a significant production system.

According to FAO, 2014 [3], family poultry production systems (FPPSs) in developing countries are diverse. Extensive systems typically involve 5–20 birds with pasture scavenging, occasional feed supplementation, limited veterinary access, and access to an indirect market. Semi-intensive systems manage 50–200 birds, featuring regular supplementation, veterinary access, and direct market access. Lastly, intensive systems exceed 200 birds, rely on commercial feed, and have access to veterinary services and markets. In peri-urban FPPSs of Argentina and Chile, 20.2, 33.0, and 46.8% were categorized as extensive, semi-intensive, and small intensive, respectively [4]. In non-intensive production systems, good husbandry and animal welfare practices (AWPs) are generally carried out, which include good density (6 birds/m^2^) [5], inspection, biosecurity, disease prevention, adequate animal health management, environmental enrichment to stimulate natural behavior, and minimizing stress [6]. Additionally, improving biosecurity during production is essential, given that the immune system of birds and their health can be affected [7].

The Campero-INTA slow-growing chicken line, developed in Argentina by the Instituto Nacional de Tecnología Agropecuaria (INTA) in the 1980s, is widely used in many FPPSs [8,9]. This line constitutes an improved breed suitable for free-range poultry farming, as usually carried out by small producers. Free-range systems improve slow-growing broilers’ nutritional, food safety, and sensory properties [10]. This breed goes through three stages: a growing stage from birth to 35 days, a development stage from 36 to 60 days, and a finishing stage from 61 days until slaughter. Slaughter occurs close to sexual maturity, which improves organoleptic characteristics and typically happens between 70 and 90 days. The estimated feed intake is 3.000 kg, and the live weight ranges from 3.000 to 3.300 kg [8,9].

Chicken coccidiosis, caused by protozoan parasites of the genus *Eimeria*, is one of the most important avian parasitic diseases, negatively impacting chicken health, performance, and productivity [11]. A recent study in FPPSs of Argentina and Chile revealed that the *Eimeria* sp. prevalence was 85.1% and that the most common species in these systems were *E. mitis* (70.3%), *E. acervulina* (62.2%), *E. tenella* (59.5%), *E. maxima* (43.2%), and *E. praecox* (32.4%), followed by *E. necatrix* (18.9%), and *E. brunetti* (5.4%) [4].

Control measures in FPPSs primarily rely on good husbandry and biosecurity practices, alongside anticoccidial drugs, typically administered through additives or water. However, environmental impacts and the emergence of resistant *Eimeria* strains have limited the use of anticoccidials, or even banned them in many countries [11]. Furthermore, the high cost of anticoccidial vaccines restricts their application in FPPSs.

Agroecology (AE) is a science and practice focused on establishing sustainable systems by integrating diverse disciplines and promoting natural processes using local resources [12]. Specifically, agroecological poultry farming offers a more sustainable and environmentally friendly alternative to conventional production, often through reduced external inputs and enhanced animal welfare practices [13].

Susceptibility to *Eimeria* infection varies among chicken breeds [14,15,16] and comprehensive information regarding Campero-INTA broilers’ susceptibility to the disease remains unknown. We have hypothesized that *Eimeria* sp. infection negatively affects productivity, and that this impact can be ameliorated through suitable chicken management, including animal welfare, together with the inclusion of a natural biopreparation. Therefore, the objective of this study was to evaluate the susceptibility of Campero-INTA chickens to four doses of the most prevalent *Eimeria* sp. based on infection parameters, i.e., intestinal lesion score (LS), oocyst output, mucosal integrity, mortality rate; productive parameters, i.e., body weight gain rate (BWG), feed conversion (FC), and the calculation of productivity index (PI). Additionally, we aimed to assess the effect of an *Eimeria* sp. infection on this chicken breed reared on AWP using either a commercial feed or an organic fermented additive, determined by both infection and productive parameters, in addition to the anticoccidial index (ACI) at two points post-infection, and the PI of the entire production cycle (75 days). This study provides foundational knowledge regarding the susceptibility to coccidiosis of the Campero-INTA chicken breed, which can be valuable for the control and prevention of this disease by applying an agroecological approach. It also represents an initial step in understanding the impact of coccidiosis in FPPSs in Argentina, promoting improved husbandry practices through a sustainability approach, enhancing animal health, and diminishing environmental pollution. Finally, this research offers valuable insights into the main microorganisms identified through metagenomic analysis of the organic fermented additive, which will be relevant for future investigations.

## 2. Materials and Methods

### 2.1. Organic Fermented Additive Preparation

Organic fermented additive was prepared as previously described Gramaglia C, 2022 [17,18]. Two bags of 10 kg filled with green vegetables and larger dry materials (sticks and stones) were used, and 20 kg/bag of cornmeal was added. In a 200 L plastic bucket, 10 L of potable water was placed to dissolve 10 kg of common sugar. Subsequently, the ingredients were mixed, and when approximately 30–40% humidity (discerned by touch) [17] was achieved, the material was transferred to a plastic drum sequentially and in thin layers to avoid air chamber formation. The drum was closed with a plastic lid and left at ambient temperature, under natural shade, to allow anaerobic fermentation. After 30 days, the organic fermented additive was used as a solid supplement to commercial food as described in Section 2.6.2. It was stored hermetically in sealed jars in a dry environment. All the materials were strictly sanitized and disinfected before use. Organoleptic properties were tested before use.

### 2.2. Metagenomic Analysis of the Organic Fermented Additive

DNA was extracted from 250 mg of the solid organic fermented material using the ADN PuriPrep-Suelo Kit (INBIO HIGHWAY^®^, Tandil, Argentina) according to the manufacturer’s instructions. The DNA concentration and quality were assessed in a P-Class P330 Nanophotometer (Implen, Munich, Germany).

DNA samples were sequenced using the Illumina platform (adapted COVIDseq kit; 2 × 150 bp reads on a NovaSeq 6000). Raw Illumina reads were subjected to quality control using Trim Galore (v0.6.10), FastQC (v0.12.1), and Kraken2 (v2.1.2). A metagenomics analysis protocol was conducted, utilizing the largest Kraken2 index database (nt_Database, downloaded in December 2024) and Kraken tools (downloaded in November 2023). Species abundance was estimated using Bracken (v2.9) [19] based on Kraken’s reports, and a comparison table was obtained using Pavian [20], which is an interactive browser application for analysing and visualizing metagenomics classification results from classifiers such as Kraken 2. Metagenomic data was submitted to the NCBI Bioproject under the Submission ID: SUB15225959 and the BioProject ID: PRJNA1245435.

### 2.3. Legal Framework

The experimental procedures were approved by the Institutional Committee for the Care and Use of Experimental Animals (CICUAE) under the numbers 27/2023 and 21/2024, ensuring animal welfare and adherence to bioethics principles.

### 2.4. Parasites

Freshly sporulated oocysts from a local isolate were used [4]. The ImageJ (version 1). JS program was used to semi-quantify the amplicons on the agarose gel and to calculate the *Eimeria* sp. relative proportions in the sample after multiplex-PCR targeting specific SCAR regions [21]. The isolation contained 37.3% of *E. acervulina*, 10.6% of *E. maxima*, 22.0% of *E. mitis*, 14.0% of *E. praecox*, and 16.1% of *E. tenella*. Parasites were maintained and propagated through passage via oral infection at 7-day-old Campero-INTA broilers. Procedures for parasite collection, purification, sporulation, and molecular identification of *Eimeria* sp. were carried out as previously described [4].

### 2.5. Animals and Management

Male and female Campero-INTA chickens were reared in safety boxes located in the Center for Agronomic and Veterinary Science Research of INTA with controlled ventilation and intergroup biosecurity protocols. Chickens vaccinated against Marek disease were fed with commercial starter feed (Table 1) from birth to 28 days, 50% *w*/*w* starter/finisher from 29 to 35 days, and a finisher feed (Table 1) from 36 to 75 days. The feed was free of anticoccidial drugs. Chicks were raised from day 1 to the end of each experiment on the floor in circular sheet-iron pens with manual feeders and drinkers, provided with sterilized shavings as bedding. The floor pens measured 0.8 m in diameter, except for groups raised with AWP, for which pens with 1.2 m diameter were used. Food and water were provided *ad libitum*. All chickens and feed were weighed weekly. The temperature and humidity of the environment and litter were controlled and recorded daily with electronic thermometers and hygrometer devices.

Professionals involved in sample collection and data acquisition, such as animal and feed weight measurements, were blinded to the experimental group assignments. Veterinarians carried out clinical evaluation and chicken monitoring weekly. On the indicated day, chickens were humanely euthanized using the cervical dislocation method.

### 2.6. Experimental Design

A completely randomized design was employed, based on the assumption that any variability would be homogeneously distributed across the groups. This design accounted for two sources of variability: the applied treatments and random error. The experimental units were randomly assigned to the various treatments. Two independent experiments were carried out.

#### 2.6.1. Trial 1

Fifty Campero-INTA chicks were weighed and randomly divided into five groups with two replicas (ten chickens per group). Each animal per replica served as the experimental unit for data collection, except for fecal samples and FC, for which each pen was the experimental unit. At 7 days old, chickens were orally challenged with freshly sporulated oocysts: low dose group (LDG) received 6000 oocysts; intermediate dose group (IDG) received 35,000 oocysts; high dose group (HDG) received 50,000 oocysts; pre-inoculated high dose group (pHDG) received a 200 sporulated oocysts on the second day after birth, and then with 50,000 oocysts at 7 days. Another group, NC, served as a non-challenged control and received the same volume of sterile phosphate-buffered saline (PBS) orally. At 6.5 days post-infection (dpi), which corresponds to 14 days of life, all chickens were euthanized (5 chicks per replica). This experiment aimed to evaluate the susceptibility of Campero-INTA to *Eimeria* infection to select the infective dose for trial 2.

#### 2.6.2. Trial 2

Two hundred male and female Campero-INTA chickens were randomly divided into five groups of five replicates. Each replicate pen served as the experimental unit for data collection, including fecal samples, FC, BWG, and necropsies. This yielded 5 data points per group, representing distinct experimental conditions. An initial allocation of 8 chickens per replicate was planned, as 4 chickens per group were euthanized for lesion evaluation during the trial. Subsequently, 10 chickens per group (2 per replicate) were euthanized for necropsies at 6.5 (15 days old) and 19.5 (28 days old) dpi. The remaining birds (4 per replica) were raised until 75 days of age, which was the entire productive pilot cycle.

Three groups were orally challenged with 35,000 sporulated oocysts per chicken at 7 days. The challenged groups were designed as follows: NTG, non-treated control; AWPG, raised under AWP; OF-AWPG, raised under AWP with the solid organic fermented additive, which was mixed with the commercial feed (Table 1) as follows: 20% *w*/*w* from birth to 28 days, 15% *w*/*w* from 29 to 35 days, and 10% *w*/*w* from 36 to 75 days the experiment. Two non-challenged, non-treated groups were named: px-NTG, raised in proximity (less than 2 m) without a physical barrier to the challenged groups; NC, non-challenged-not treated control, raised physically separated.

The AWP included a lower density per pen, achieved by using larger pens of 1.2 m yielding 6.6 vs. 12.3 birds/m^2^; higher beddings (20 cm vs. 10 cm high); strict hygiene with a weekly frequency vs. monthly; control of litter humidity. Elevated elements for pecking and perching were included in the pens, allowing chicken behavioral development. This experiment aimed to assess the effect of good husbandry practices, including animal welfare and the organic fermented additive mixed with a commercial feed.

### 2.7. Oocyst Quantification

Fresh fecal samples were collected directly from pens at the beginning of the study every three days up to the challenge (day 7) and daily up to the end of the trials. Samples were kept at 4 °C and processed within 3 days. Fecal samples for each replica were pooled, and oocysts per gram of feces (OPG) per group were counted in triplicate as previously described [4]. Briefly, 5 mg of feces pooled was homogenized in a mortar in 70 mL of saturated saline solution, filtered through a metal strainer, and stirred for 5 min. The McMaster 4-chamber slide was filled with the saline suspension (0.5 mL/chamber) and allowed to rest for 5 min before counting. Floating *Eimeria* sp. oocysts were microscopically recognized by their morphology and counted. OPG was calculated as follows:

∑ Nr. of oocysts in each chamber × 70 mL × 5 g ^−1^ × 2 mL ^−1^ = oocysts × 7 g

Negative OPGs corresponded to the absence of oocysts in the four chambers.

### 2.8. Infection Parameters: Lesion Score, Microscopic Observation, Histopathological Analysis, and Clinical Signs of Coccidiosis

Necropsies were performed at 6.5 on all chickens (N = 5 per replica, 10 per group) in trial 1 and at 6.5 and 19.5 dpi in trial 2 (N = 2 per replica at each timepoint, 10 per group). The intestine was extracted, and the duodenum, jejunum, ileum, and caecum were separated, washed with sterile PBS 1X, and macroscopically examined. Each intestinal region was opened longitudinally, and the luminal contents were removed and washed with sterile PBS. Every intestinal region was analyzed for LS using the Johnson and Reid scoring [22], where 0 means absence of lesions and 4 is the maximum. The duodena were fixed in 10% formaldehyde for histopathological analyses and stained with hematoxylin and eosin (HE). HE-stained slides were scanned and assessed for the villus height (VH), crypt depth (CD), and the villus height–crypt depth ratio was calculated. Ten measures per slide were made using the QuPath program 0.5.1. The analyses were performed blindly. In trial 1, 5 animals per replica (2 replicates per group) were included (n = 5; N = 50), and in trial 2, 2 birds per replicate were included (n = 2; N = 50).

Experienced veterinarians monitored the chickens weekly throughout each experiment for health status and clinical signs such as anorexia, diarrhea, bristly feathers, and abnormal behavior.

### 2.9. Production Parameters: Body Weight Gain, Feed Conversion, Productivity Index, and Anticoccidial Index

Chickens were weighed individually at the beginning and weekly up to the end of the experiments (14 and 75 days old in trials 1 and 2, respectively). The calculation and data analysis were carried out independently by three professionals. BWG was calculated as follows = final body weight − initial body weight. The relative BWG (rBWG) = corresponding BWG of the treated group/BWG of the NTG control group.

The feed of each pen was weighed weekly, and feed intake was calculated as the difference between the weight at the beginning and the end of the week. FC was calculated as the ratio between the mass (g) of feed intake and the BWG (g) per group weekly. In trial 1, 5 animals per replica (two replicates per group) were included (n = 5; N = 50), and feed intake consisted of 2 measurements per group (n = 1; N = 10). In Trial 2, BWGs up to 14 days were performed in 8 chicks per replica, 5 replicates per group (n = 8, N = 200); up to 35 days: 6 chickens per replica (n = 6, N = 150); until 75 days in 4 chicks per replica (n = 4, N = 80). The experimental unit was each pen for the feed intake measurements; thus, 5 replicates per treatment group were used (n = 1; N = 25).

PI was calculated as follows: BWG × relative survival rate/FC × 10 [14]. Relative survivals were relativized with negative control (NC) in trial 1 and with the non-treated group (NTG) in trial 2.

Anticoccidial efficacy was measured at 6.5 dpi (14 days old) and 19.5 dpi (27 days old) in trial 2 by the ACI, calculated as follows: (relative ratio of BWG + survival rate) − (LS × 10 + oocyst value) [23]. ACI values below 120 mean no anticoccidial efficacy; between 120 and 140, mild or slight; between 140 and 160, moderate; between 160 and 180, good; above 180, high anticoccidial efficacy [24]. Oocyst ratios (ORs) were calculated as OPG in the treated group/OPG in the non-treated group x 100 and then converted to the oocyst index values (OI) of 0, 5, 10 and 20, which corresponded to ORs of 0–1, 2–25, 26–50, and 51–75%, respectively [25].

### 2.10. Statistical Analysis

The mean values of the quantitative variables for each experimental group were analyzed using a one- or two-way Analysis of Variance (ANOVA) as appropriate, followed by Tukey’s post-test. In turn, compliance with the assumptions of homoscedasticity and normal distribution was previously evaluated using the Levene test and the Shapiro–Wilk test, respectively. Furthermore, in the event that the parametric assumptions were not met, the nonparametric Kruskal-Wallis test was applied, followed by Dunn’s post-test. A correlation analysis was performed using Pearson’s coefficient (normal distribution). Values close to 1 are considered strongly associated. Results were expressed as mean ± standard deviation (SD), with a significance level set at *p* < 0.05 to indicate statistically significant differences. Data was analyzed using GraphPad 5 Prism V.5.0.1 software.

The sample size was decided based on a combination of statistical considerations and practical constraints. A priori sample size calculation (Trial 1) was performed considering a minimum weight gain of 50 g with a standard deviation (SD) of ±40 g. An alpha risk of 0.1 and a beta risk of 0.2 were accepted in a two-sided test, with an estimated follow-up loss rate of 11.7%. Based on these parameters (and assuming a formula for comparing two means), a minimum of 10 animals per group was calculated. Adjustment based on observations of trial 1, where no mortality was observed, the loss percentage was adjusted to 8% in trial 2. Recalculating the sample size with this new rate resulted in a minimum of 8 animals per replica. These sample sizes were used as a minimum to allow statistical analyses and to align with the 3R principles, taking animal availability into account.

## 3. Results

### 3.1. Trial 1: Susceptibility of Campero-INTA to Eimeria-Infection

#### 3.1.1. Oocysts Excretion, Microscopic Lesions, and Clinical Signs of Coccidiosis

OPGs remained negative until 4 dpi, and significant differences were observed between the NC and the challenged groups (*p* < 0.05). The maximum excretion was observed between 6 and 7 dpi, except for pHDG, which had its maximum excretion at 5 dpi.

**Figure 1:**
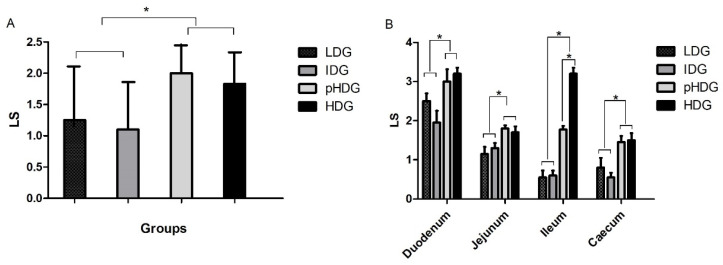
Lesion score (LS) in the intestines of challenged groups at 6.5 days post-infection. (**A**) Mean (N = 10 per group) of LS for the four intestinal regions with their standard deviation (SD) are depicted. (**B**) Mean of LS of each intestinal region with the SD. Groups: low dose (LDG) received 6000 oocysts; intermediate dose (IDG) received 35,000 oocysts; high dose (HDG) received 50,000 oocysts; pre-inoculated high dose (pHDG) received 200 oocysts on the second day after birth, then 50,000 oocysts at 7 days. In the non-challenged control, no lesions were found. Asterisks show significant differences (*p* < 0.05).

The average LS from the fourth intestinal regions showed medium and low scores (ranging from 2 to 3) in all challenged groups (Figure 1A) with significant differences compared to NC (Tukey’s test, *p* < 0.05). LDG and IDG showed a significant (Tukey’s test, *p* < 0.05) difference between HDG and pHDG (Figure 1A), and IDG exhibited the lowest LS. The highest LS were found in the duodenum of all challenged groups with scores of 2.5, 1.9, 3.0, and 3.2 in LDG, IDG, pHDG, and HDG, respectively (Figure 1B).

**Figure 2:**
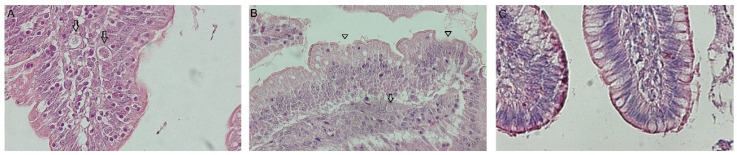
Histological sections of the duodenum stained with hematoxylin and eosin (HE). (**A**) *Eimeria*-asexual stages (arrows) in IDG (images are representative). (**B**) Histological changes with affected villi (triangles) and *Eimeria*-asexual stages (arrows) in IDG. (**C**) Negative control with normal morphology. Total magnification 400×.

Villi damage and asexual stages of the parasite were observed in HE stains in all challenged groups (Figure 2A,B), and no particularities were observed in NC (Figure 2C). Additionally, changes in the VH (*p* < 0.05) were observed in the duodenum of challenged groups. The VH–CD ratio showed an alteration of the normal morphology of the duodenum among the challenged groups and the not in NC (Tukey’s test, *p* < 0.05), with HDG being the most affected (2.88) and LDG the least (3.78) (Table 2).

Whereas clinical signs of coccidiosis were absent in LDG and NC, mild diarrhea was present in IDG and pHDG, and HDG exhibited lethargy, diarrhea, and ruffled feathers. Importantly, no chicken death was observed.

#### 3.1.2. Body Weight Gain, Feed Conversion, and Productivity Index

**Figure 3:**
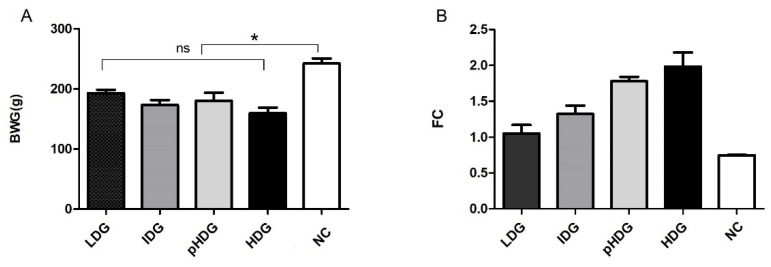
Productive parameters at 6.5 days post-infection (dpi). (**A**) Body weight gain (BWG). (**B**) Feed conversion (FC). NC is the non-challenged control group. Asterisk means significant difference (*p* < 0.05), and ns means no significant differences.

BWG was impaired in all challenged groups, showing significant differences (*p* < 0.05) with the non-challenged, post-inoculation, but no significant differences were observed among the three challenged groups (*p* < 0.05) (Figure 3A). Additionally, a negative correlation between BWG and LS was found (Pearson coefficient: −0.927), i.e., the worse the BWG, the higher the LS. Conversely, a strong positive association between VH and BWG (Pearson: 0.602), meaning that a shorter villi height implies a lower BWG. A strong negative association between VH and LS (Pearson: −0.685) was also found; in other words, the lowest VH, the highest LS.

The lowest FC was found for the NC, followed by LDG, IDG, pHDG, and HDG (Figure 3B). Accordingly, the PI was higher in the non-challenged than in the challenged groups, and it decreased as the dose increased; therefore, the lowest index was obtained for the groups pHDG and HDG (Table 2).

### 3.2. Trial 2: Effect of Good Husbandry Practices and Organic Fermented Additive in Campero-INTA Broilers

#### 3.2.1. Organoleptic Properties and Metagenomics of Organic Fermented Additive

Before use, the sensory and physical characteristics of the organic ferment additive were observed. It possessed a whitish mycelium on the surface, yeast odor, a light brown color, 30% moisture content (approx.), and a pH = 5.

The results showed that the dominant bacteria were from the Firmicutes phylum, Lactobacillales order, and the genera *Lentilactobacillus* and *Lacticaseibacillus* (Table S1). At the species level, the most abundant bacteria were *Lentilactobacillus buchneri* (62.04%), followed by *Lacticaseibacillus paracasei* (11.93%), *Schleiferilactobacillus harbinensis* (3.31%), *Lactiplantibacillus plantarum* (*ex-Lacticaseibacillus plantarum*) (1.14%), and *Streptomyces cyaneogriseus* (0.57%). On the other hand, the most abundant eukaryote was *Zea mays* (2.42%).

#### 3.2.2. Clinical Signs of Coccidiosis and Mortality

While clinical signs of coccidiosis were not observed in px-NTG and NC, the rest of the groups showed mild diarrhea without noticeable differences between groups, as was observed in trial 1 for IDG. No mortality was observed in the whole trial.

#### 3.2.3. Oocysts Excretion, Lesion Scoring, and Microscopic Lesions

**Figure 4:**
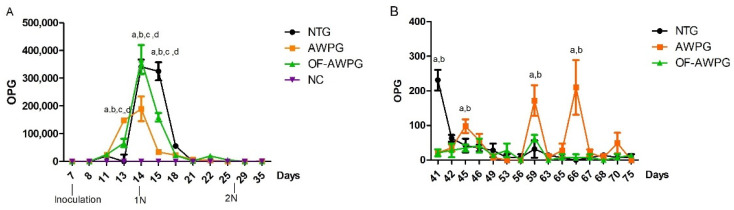
Oocyst per gram of feces (OPG) during trial 2. (**A**) OPG during the growing phase (35 days). Inoculation is indicated at day 7, and necropsy 1 (1N) and 2 (2N) carried out at 6.5 and 19.5 dpi are also depicted. There were significant differences (*p* < 0.05) between all groups at 13, 14, and 15 days, which are indicated with lowercase letters. (**B**) OPG during the regrowing phase up to the slaughter time (75 days). Significant differences (lowercase letters) were found at 41 days between NTG and the rest of the groups, at 45, 59, and 66 days between AWPG and the rest of the groups. Groups: NTG is the non-treated control (black circle); AWPG is the group raised under animal welfare practices (AWP) (yellow square); OF-AWPG, was raised under AWP with organic fermented additive (green triangle); and NC is the non-challenged-not treated control raised physically separated (purple upside-down triangle).

OPG in NC remained negative throughout the experiment, as expected. For the other groups, OPG was negative until 4 dpi and then started shedding, except px-NTG, which started shedding at 8 dpi, when the chickens were 15 days old. The maximum excretion occurred at 7 dpi (14 days) in OF-AWPG (OPG = 367,500) and NTG (341,600) (Figure 4A), and there were significant differences between NTG and AWPG, NTG and OF-AWPG, and between AWPG and OF-AWPG (*p* < 0.05). Interestingly, in OF-AWPG, oocyst shedding diminished drastically and remained very low from 13 dpi (20 days) up to the end of the trial (Figure 4A,B). From 13 dpi (20 days), OPGs remained very low in all groups; however, NTG increased significantly the oocysts excretion at 41 and 59 days, and AWPG at 45, 59, 66, and 70 days (Figure 4B).

**Figure 5:**
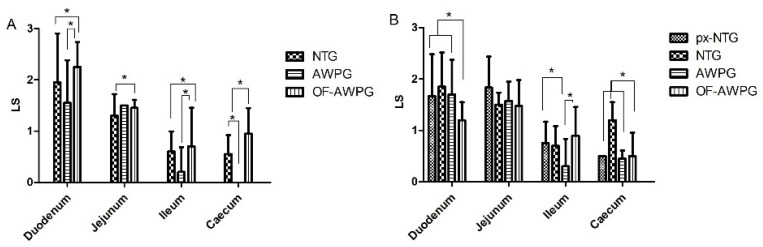
LS at 6.5 dpi (**A**) and 19.5 dpi (**B**). Asterisks indicate significant differences (*p* < 0.05). Cecal lesion in AWPG was 0 at 6.5 dpi.

Significant differences in LS were observed among the challenged groups when compared to the NC (*p* < 0.05). LS at 6.5 dpi showed a significant difference in the duodenum, ileum, and caecum between AWPG and OF-AWPG and between NTG and OF-AWPG (Figure 5A), while in px-NTG and NC, scores remained 0. The highest LS values were observed in the duodenum (mean range: 1.50–2.25), especially in OF-AWPG (mean LS: 2.25 ± 0.49). LS values at 19.5 dpi were lower than at 6.5 dpi, the duodenum being the most affected (Figure 5A). For the case of px-NTG that became infected later, no significant difference between groups was observed (*p* > 0.05) (Figure 5B). Interestingly, significant differences were observed in the duodenum at 19.5 dpi between px-NTG, NTG, AWPG, and OF-AWPG. (Figure 5B). Additionally, LS in the ileum and ceca showed some differences in AWPG, OF-AWPG, and NTG (Figure 5B).

Histopathologic analysis showed that mild occurrences of different asexual forms of *Eimeria* in the duodenum, jejunum, and ileum, with the most significant presence in the duodenum at 6.5 dpi. The accompanying pathological changes were minimal, with mild lymphocytic infiltration. At this time point, no significant difference in villus height was observed between the treated and non-treated groups (Table S2). However, at 19.5 dpi, NTG exhibited significantly shorter villi (871.88 ± 157.7 µm, *p* < 0.05) compared to AWPG (989.61 ± 236.7 µm) and OF-AWPG (964.19 ± 236.9 µm). From 6.5 to 19.5 dpi, villus height increased by 13.15% and 13.28% in the AWPG and OF-AWPG groups, respectively, while it decreased by 6.03% in the NTG group. Although crypt depth did not significantly differ among groups, OF-AWPG showed the most favorable villus height–crypt depth ratio (3.25) compared to NTG (2.74) and AWP (3.06) (Table S2).

#### 3.2.4. Body Weight and Feed Conversion

At 6.5 dpi, the BWG of NTG (167.33 ± 13.18), AWPG (178.84 ± 10.54), and OF-AWPG (164.66 ± 7.55) were significantly lower (*p* < 0.05) than the NC (249.10 ± 14.72) and the px-NTG (215.00 ± 8.35) that did not become infected at this timepoint (Table S3). FC showed the same pattern, being 1.9, 1.7, and 1.4 for NTG, AWPG, and OF-AWPG, respectively, and 2.3 and 2.1 for NC and px-NTG, respectively. At 19.5 dpi, BWG was 515.00 ± 29.70, 535.69 ± 44.09, 520.54 ± 32.36, 619.83 ± 38.22, and 606.80 ± 55.18 for NTG, AWPG, OF-AWPG, px-NTG, and NC, respectively.

Because individual parameters failed to accurately indicate the anticoccidial effect, the ACI was calculated at 6.5 and 19.5 dpi for the two treated groups and the NTG. The latter group showed a moderate ACI at both 6.5 (14 days) and 19.5 dpi (27 days). Interestingly, whereas good efficacy at both dpi was found in the AWPG, a moderate ACI was found at 6.5 dpi, and high efficacy at 19.5 dpi was found for OF-AWPG (Table 3).

**Figure 6:**
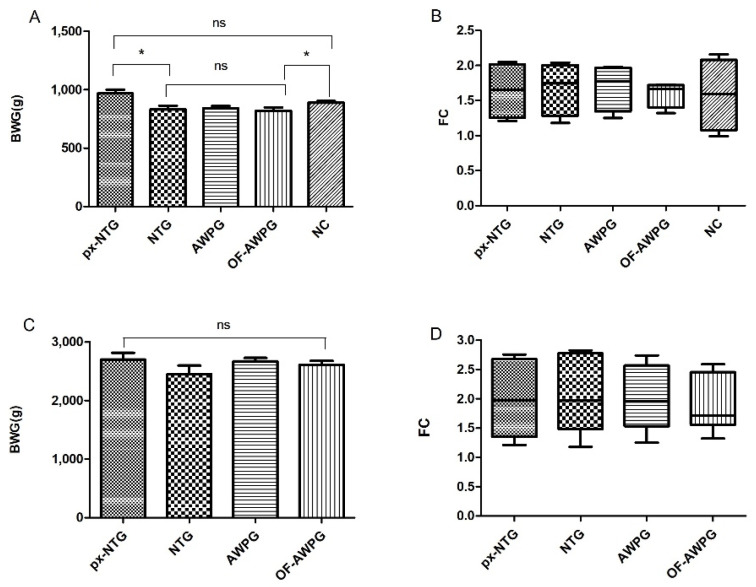
Production parameters in the growing and regrowing stages. The average ± SD of BWG (**A**) and FC (**B**) at 35 days, and BWG (**C**) and FC (**D**) at 75 days. Asterisks show significant differences, ns: no significant difference. The px-NTG is the non-treated group raised in proximity to the challenged groups.

Productive parameters at the end of the growing phase (35 days) showed that BWG was significantly different (*p* < 0.05) between px-NTG and NC and the three challenged groups (Figure 6A), but no significant differences were observed between px-NTG and NC, nor among the three challenged groups (Tukey’s test: *p* > 0.05) (Figure 6A).

Regarding FC, no notable differences were observed at this growth phase between groups (Figure 6B).

At the slaughter time (75 days), the BWG and FC for all groups were similar without significant differences (*p* > 0.05) (Figure 6C,D); however, the FC of OF-AWPG was 14.52% lower than the NTG (2.40 vs. 2.80) (Figure 6D). Daily weight in the growing (1–35 days) and regrowing (36–75) periods is shown in Table 4, and no significant differences were observed among groups (*p* < 0.05). Analyzing these parameters individually might give a limited standpoint; in this regard, the IP offers a holistic perspective of performance, comprehensively evaluating the production cycle. The highest IP was obtained for OF-AWP (108.79), being 24.44% better than NTG (87.43) (Table 5).

## 4. Discussion

Family farming plays a crucial role in resource-limited rural and peri-urban households worldwide [3]. In Argentina, it plays a significant role in food security, while simultaneously contributing to environmental sustainability and promoting rural development, improving families’ quality of life. Consequently, advancing our understanding of diseases, especially coccidiosis, is essential to improve productivity in FFPS. In this sense, to assess the effect of *Eimeria* sp. doses in the Campero-INTA slow-growing broiler breed, three doses of *Eimeria* sp. and a pre-inoculation on the second day were evaluated and tested. Clinical coccidiosis was reproduced, and clinical signs, including mild diarrhea, lethargy, and ruffled feathers, were observed in addition to low LS found in all challenged groups (Figure 1A). These signs are typically associated with *Eimeria* species exhibiting moderate to low pathogenicity, which aligns with the predominant species present in the inoculum, i.e., 37.3% of *E. acervulina*, 10.6% of *E. maxima*, 22.0% of *E. mitis*, 14.0% of *E. praecox*, and 16.1% of *E. tenella*. *E. acervulina* and *E. maxima* are considered of moderate pathogenicity and represent 47.6% of the total, and *E. mitis* and *E. praecox* are considered of low pathogenicity [26], representing 36% of the total species. It is also worth noting that *E. praecox* parasitizes the duodenum [27]. Thus, the higher score found in the duodenum may be explained by the concomitant presence of these two species contained in the inoculum. *E. tenella* was in low proportions (16.1%) within the mix of species, which may explain the low score found in the caecum (Figure 1B).

Furthermore, *E. acervulina* was the predominant species in the inoculum and parasitizes the duodenum, causing the shortening of villi and increasing crypt depth [28]. In this study, affected villi and asexual parasite stages were observed (Figure 2A,B), and it was also demonstrated that deeper crypts and shorter villi were found upon *Eimeria* challenge. The worst VH–CD ratio was 2.88 in HDG, indicating more damage than the other groups, probably affecting gut functionality. This aligns with previous findings in Hy-line Brown layers [29], Ross 308 broilers [30], and Yellow broilers [31], which indicates that *Eimeria* infection led to deeper intestinal crypts, decreasing the VH–CD ratio, demonstrating a slower enterocyte turnover. However, Belote et al., 2023 [32], have concluded that in broilers challenged with field *Eimeria* oocysts, the augmented villi size does not necessarily correlate with improved intestinal functionality when internal immune processes drive this enlargement. In contrast, the NC group showed that the best VH–CD ratio was 6.12. The ratio identified by De Verdal et al., 2010 [33] was 7.73 in the duodenum of a broiler line at 23 days, implying a greater rate of epithelial cell turnover in the proximal small intestine.

This study revealed that all doses impaired the BWG compared to the non-challenged control (Figure 3A), despite the moderate level of coccidiosis induced. Conway et al. [34] established a correlation between lesion score and weight gain, demonstrating an inverse relationship: as LS increases, weight gain decreases, and vice versa. While they also noted that macroscopic lesion size does not fully reflect infection severity, our study partially aligns with this observation. Statistically significant differences in LS were observed between the challenged and non-challenged groups and between the low/intermediate and the highest doses, indicating that the impact of coccidiosis becomes more severe as the dose increases. *E. acervulina* is considered a subclinical species, causing malabsorption syndrome and has a negative impact on productive parameters [35]. This potentially explains the decrease in the BWG and diarrhea observed in challenged chickens.

The present study also sought to replicate the rearing conditions observed in Argentinian FPPS [4], including partial bed replacement after each production cycle. Given the high *Eimeria* prevalence previously observed (85%) [4], this might suggest a low dose of infective oocysts in the pens from the first days of age. Consequently, we have included a pre-inoculated group, pHDG, to assess whether a small infective dose mimicking a pre-immunization with very low doses could attenuate the impact of coccidiosis. In this trial, we did not observe a substantial attenuation of coccidiosis in terms of lesions, weight gain, or FC. However, analyzing these parameters individually might give a limited standpoint; in this sense, the IP offers a holistic perspective of performance. The PI of pHDG was higher than HDG (Table 2), suggesting that pre-inoculation could attenuate the coccidiosis effect. Nevertheless, further research is needed to establish if a low dose administered on the second day of a broiler’s life has a detrimental health effect.

As expected, the highest PI was obtained for the non-challenged group, indicating that even low doses, mainly containing *Eimeria* sp. of low pathogenicity, have a significant impact on production parameters, as reported previously [36,37,38]. The groups receiving low (16.83) and intermediate (14.18) doses showed the best productivity indexes (Table 2). Based on the significant differences in LS and in the substantial numerical difference in PI between high (9.47 and 7.49) and intermediate (14.18) doses, and to minimize suffering and mortality according to animal welfare and the 3Rs principle, the intermediate dose would be suitable to evaluate the effects of sustainable control measures in this chicken breed with the mix of frequent *Eimeria* sp. of low and moderate pathogenicity. Moreover, it has been our observation [4] that severe coccidiosis is uncommon in these production systems. Still, even mild cases harm productivity. Consequently, we opted for a dose of 35,000 sporulated oocysts to induce a moderate coccidiosis; selecting this infective dose would accurately reflect the impact of coccidiosis.

Little information is available in the literature regarding the susceptibility of slow-growing broiler breeds to *Eimeria* infection. Sakkas et al., 2018 [39], investigated whether the growth rate in broilers influences resistance and tolerance to *E. maxima*, concluding that, given their experimental conditions and a suitable nutritional diet, line differences in productive performance did not affect host responses to *E. maxima.* Swinkels et al., 2007 [40], have evaluated the immune response to *E. acervulina* infection in fast and slow-growing chicken breeds. They demonstrated that, despite the different growth rates, control and infection differed in both chicken lines due to differential immune response (CD8(+) T). Therefore, this trial addresses a critical health issue for poultry farming by providing the first assessment of coccidiosis impact using the most frequent *Eimeria* sp. found in FFPS on the Campero-INTA slow-growing chicken line. By deepening our understanding of this parasitic disease, we aim to facilitate the development of sustainable and feasible control, potentially extending its applicability to other slow-growing breeds worldwide.

To improve the productivity of FFPSs, which are affected by coccidiosis as demonstrated in the first trial, we sought solutions tailored to their production systems. Recognizing that biosecurity and animal welfare are crucial factors in maintaining chicken health and considering the potential for family producers to self-produce natural ferments, this study aimed to evaluate the impact of these approaches. Furthermore, a previous survey carried out on Argentine and Chilean FFPS revealed that elements to reduce chicken stress are frequently employed (paper in progress). Thus, assessing the effect of these implemented animal welfare practices was crucial. For the AWPG, larger pens were used to maintain an optimal density of 6 birds per square meter. The beds were deeper and kept dry, feeders and drinkers were cleaned more frequently, and enrichments were provided for pecking and perching. Chickens receiving the fermented feed additive were raised under these conditions (OF-AWPG). The untreated (NTG), non-challenged, non-treated (NC), and the group not treated and reared in proximity, the less than 2 m (px-NTG) groups, were reared at a density of 12 birds per square meter, with shallow, damp beds, and without environmental enrichments. Results showed that px-NTG became infected as soon as the challenged groups began shedding oocysts, emphasizing the contagious nature of coccidiosis when there are no physical barriers or distance.

Histopathological findings and LS at 6.5 dpi aligned with previous observations in trial 1 at the same intermediate dosage (35,000 sporulated *Eimeria* oocysts/chicken), where moderate coccidiosis was induced. Consequently, NTG exhibited moderate anticoccidial efficacy (Table 3). Notably, while duodenal villi height (VH) increased from 6.5 to 19.5 dpi in AWPG (13.28% recovery) and OF-AWPG (13.13% recovery), it decreased in NTG (6.03% impairment). Furthermore, epithelial cell turnover and nutrient absorption were more negatively impacted in NTG (VH–CD ratio of 2.74) compared to AWPG (3.06) and OF-AWPG (3.25) at 19.5 dpi (Table S2). ACI showed that animal welfare management has good efficacy both at 6.5 and 19.5 dpi in the AWPG. On the other hand, the group that received the organic fermented additive (OF-AWPG), reared under the same husbandry conditions, showed a moderate ACI at 6.5 dpi, the same as the NTG. However, at 19.5 dpi (27 days old), the index improved substantially due to a significant decrease in oocyst excretion (Figure 4A,B), reaching high efficacy. It can be speculated that the ferment modulates the intestinal microbiota, thus stimulating the immune system, both of which are still developing at 6.5 dpi (14 days). It was previously demonstrated that in avian microbiomes, age played a major role in the composition and richness of the bacterial community. While major shifts occurred from day of hatch to day 14, the main diversity was found at 28 days in the White Leghorn laying chicken [41]. It is also possible that it is necessary to adjust the proportion of the organic fermented additive and feed during the different growth stages, and this was not evaluated in this trial.

Metagenomic analysis showed that *Zea mays* was the main eukaryotic organism present in the organic fermented additive. The inclusion of corn flour as a constituent of the ferment explained its presence, supporting the methodology used. Additionally, the results indicated that *L. buchneri* was the dominant prokaryote. This lactic acid bacteria (LAB) is utilized in Europe as a silage additive to enhance fresh plant material preservation and is considered safe for animals, consumers, and the environment [42]. The fermented additive was readily accepted by chickens, which kept a good health status throughout the experimental trial, despite being challenged with *Eimeria* sp. Thus, the predominant presence of *L. buchneri* in the organic fermented additive could contribute to its preservation and good acceptance during rearing. Metagenomic data also revealed the presence of *L. paracasei*, *S. harbinensis*, *L. plantarum,* and *S. cyaneogriseus*. These LAB have recently gathered significant research interest due to their roles in fermentation and probiotic health benefits when consumed in adequate amounts [43,44,45]. *L. paracasei* supplementation in laying hen diets has demonstrated improvements in egg freshness, antioxidant capacity, gut microbiota modulation, and immunity [46]. Furthermore, *L. paracasei* has been proposed as a potential broiler growth promoter, likely through gut microbiota modulation [47]. In the case of *S. harbinensis*, the bioactive activity of its secondary metabolites, i.e., exopolysaccharides, has shown beneficial effects on gut microbiota [48] or in lowering cholesterol [49]. As mentioned above, this study demonstrated a substantial improvement in the ACI at 19.5 dpi compared to 6.5 dpi in the group receiving the organic fermented additive (Table 3); the improvement of ACI was mainly due to the drastic reduction in oocysts output, which remained very low up to the end of the trial (Figure 4A,B). Furthermore, the FC at the slaughter time was superior in this group compared to the others (Figure 6D). It is possible to speculate that the content of LAB in the ferment might promote gut microbiota, ameliorating the coccidiosis impact. Despite the low abundance in the ferment of *L. plantarum*, it has been recently shown that this LAB reduced Salmonella Typhimurium counts in the feces and cecal contents of infected broilers, suggesting a probiotic activity [50]. Interestingly, it was demonstrated that *S. cyaneogriseus* is a thermotolerant producer of nemadectin, which is used as an insecticide and helminthicide in animal health [51,52].

While further investigation is needed to definitively attribute these findings to the potentially beneficial properties of the LAB present in the ferment and to characterize the microbial community deeper and determine the optimal ferment dosage, these results suggest that the implementation of animal welfare practices in slow-growth broiler rearing and the supplementation with the organic fermented additive have a potent coccidiosis-preventive effect. It is important to note that this homemade ferment followed strict hygiene procedures [17,18]. Despite the benefits of probiotics, their implementation by stakeholders should consider the importance of sanitary safety measures to avoid contamination with pathogenic microorganisms or mycotoxins [53]. Thus, strict hygiene measures, safe raw materials, and sanitary controls need to be applied before adding to animal feed.

The growth observed in all treatments during both periods (growing and regrowing) aligns with the expected performance for this slow-growing line [54]. Nevertheless, this work yielded a slightly greater average daily weight gain for female chickens compared (Table 4) to previous reports, where in the regrowing period, it was 52.7 ± 0.80. This difference could potentially be explained by variations in the rearing system and/or the diet provided between the current study and those reported, or by different susceptibility to coccidiosis between male and female chicks, although further investigation is required to elucidate this finding. Finally, it was shown that all groups except the untreated one (NTG) reached the slaughter weight (Figure 6C). The px-NTG raised without AWP and treatment acquired infection during the second week of age, which indicates that the age of infection in this slow-growing chicken line could be key to allowing weight recovery, indeed supporting the purpose of its development [9]. However, since in FFPS the high prevalence was found [4], chickens probably acquire infection very early. Thus, weight recovery is unlikely to occur on farms. Additionally, the rapid infection of this group reinforces the confirmation that it is a highly contagious disease in the absence of adequate biosecurity measures, and the impact can vary depending on the timing of the infection, the dose, and the pathogenicity of the species. Since no significant differences in productive parameters were found between the px-NTG and the NC group at the final of the growing phase (Figure 6A,B), we decided to continue the trial with the remaining groups for feasibility and economic reasons, aiming to evaluate the impact of the treatments across the entire pilot production cycle.

Although no significant differences were observed between NTG and OF-AWPG, a 14.52% improvement in FC was obtained in OF-AWPG (Figure 6D). Furthermore, the PI for the pilot experimental production cycle (75 days) for OF-AWP was 24.44% higher than the NTG (108.8 vs. 87.43, Table 5). This could positively impact economic gain by decreasing feed expenses, which constitute 80% of the productive costs. However, deeper economic studies are needed to elucidate this issue.

According to FAO, 2013 [55], Ahmed et al., 2021 [56], and Portillo Salgado and Vazquez Martinez, 2019 [57], rural communities in Asia, Africa, and Latin America exhibit a gender-based division of labor in family poultry farming. Women are often involved in management but excluded from decision-making, frequently being responsible for raising poultry and consequently facing greater chemical and biological risks from animal handling than men. Simultaneously, animal husbandry by women is often characterized by good care and consideration for animal welfare. Furthermore, the commercialization of their production serves as a tool for empowerment in some rural communities [58]. Therefore, given that women are essential to family poultry production, significantly contributing to food security, domestic economy, and community development, the potential implementation of this AE approach could benefit rural women.

Despite the number of chickens per group in both trials being small due to limitations in obtaining the chickens and the availability of physical space for carrying out the trials, the results are relevant and consistent, having been obtained through rigorous and independent analyses. The collected data was performed blinded, which strengthens its reliability. Although further studies are needed to obtain more insight, the new evidence described in this study is valuable for a breed highly used in FFPSs. This study addresses a critical health challenge by providing the first assessment of coccidiosis’s impact on the Campero-INTA slow-growing chicken line. By deepening our understanding of this parasitic disease, we aim to facilitate the development of sustainable and feasible control strategies that can benefit farmers generally, with a specific focus on solutions accessible and beneficial for women poultry farmers, potentially extending their applicability to other slow-growing breeds worldwide.

## 5. Conclusions

The present work demonstrates that the induction of a mild to moderate infection with the most prevalent local *Eimeria* spp., mainly of moderate pathogenicity, causes a decrease in productive parameters of the Campero-INTA slow-growing chicken breed, reflected in a lower PI compared to the unchallenged group. Additionally, the second trial showed that an AE approach, tailored to the needs of small-scale farmers, could be highly effective in controlling this prevalent parasitic disease and improving the productive parameters, the ACI, and the PI. By diminishing the economic impact of coccidiosis, it could, consequently, enhance the quality of life of family farmers, especially women, thus contributing to the reduction of rural poverty. This strategy would offer environmental and economic advantages for small producers, rural communities, and society in general. This work constitutes the first report on the integrated application that combines an organic fermented additive with animal welfare practices for coccidiosis control. This approach enhances animal comfort through environmental enrichment within an animal welfare framework for the control of this parasitosis, and also presents an opportunity to minimize the environmental impact of currently used anticoccidial drugs. Although it is necessary to increase the number of animals to obtain more reliable data, these preliminary results support the proposed hypothesis.

Finally, metagenomic analysis provided valuable information about the main microorganisms that compose the ferment generated under experimental conditions for the present study and provides a solid foundation for future studies on probiotic applications.

## Figures and Tables

**Table 1:** List of feed ingredients employed in this work.

Ingredients	Rations	
Name	Starter (1–28 Days) *	Finisher (36–75 Days) *
Monocalcium phosphate 22.7%	1.450	0.840
Soybean expeller	41.900	28.400
Yellow corn	54.050	68.060
Premix INI-TERM 1.4% CLADAN	1.400	1.400
Oyster Shell	1.200	0.800
Soybean oil	NA	0.500

* Between 29 and 35 days, starter and finisher feed were mixed at 50% *w*/*w*. Abr.: NA: not added.

**Table 2:** Infection and productive parameters.

Groups	LS	VH (µm)	CD (µm)	VH–CD	BWG (g)	FC (g)	PI
LDG	2.50 ± 0.62 ^a^	984.49 ± 143.77 ^a^	264.41 ± 82.65 ^a^	3.78 ^a^	176.75 ± 58.74 ^a^	1.05 ± 0.17	16.83
IDG	1.95 ± 1.00 ^a^	927.16 ± 97.84 ^a^	285.64 ± 71.65 ^a^	3.24 ^a^	167.33 ± 63.47 ^a^	1.18 ± 0.27	14.18
pHDG	3.00 ± 1.00 ^a^	946.66 ± 52.76 ^a^	298.41 ± 68.26 ^a^	3.17 ^a^	168.50 ± 56.68 ^a^	1.78 ± 0.08	9.47
HDG	3.20 ± 0.63 ^b^	860.31 ± 137.13 ^b^	286.12 ± 42.22 ^a^	2.88 ^a^	158.25 ± 47.24 ^a^	1.98 ± 0.28	7.99
NC	0.00 ± 0.00 ^c^	999.50 ± 70.39 ^a^	163.23 ± 81.16 ^b^	6.12 ^b^	290.00 ± 63.68 ^b^	0.75 ± 0.02	29.29

Abr.: VH: Villus height of duodena; CD: crypt depth of duodena; PI: productivity index. Different superscript letters indicate significant differences (*p* < 0.05).

**Table 3:** Anticoccidial index (ACI) at 6.5 and 19.5 dpi.

Groups	rBWG	S%	LI	OI	ACI	Efficacy
6.5 dpi						
AWPG	106.88	100	9.00	20	177.9	Good
OF-AWPG	98.40	100	12.80	40	145.4	Moderate
NTG	100.00	100	11.00	40	149.0	Moderate
19.5 dpi						
AWPG	104.02	100	10.10	20	173.9	Good
OF-AWPG	101.07	100	11.00	10	180.6	High
NTG	100.00	100	13.20	40	146.7	Moderate

Abr. rBWG: relative body weight gain; S% survival rate, LI: lesion index; OI, oocyst index. ACI efficacy: No efficacy < 120; Mild: 120–140; Moderate: 140–160; Good: 160–180; High: >180 [24].

**Table 4:** Daily weight in the growing (1–35 days) and regrowing (36–75) period.

Period (Days)	px-NTG			NTG			AWPG			OF-AWPG			NC		
	M	F	A	M	F	A	M	F	A	M	F	A	M	F	A
1–35	12.2 ± 0.8	11.1 ± 0.9	11.6 ± 0.9	10.2 ± 1.3	9.8 ± 0.7	10.0 ± 1.0	10.9 ± 1.2	9.5 ± 0.8	10.2 ± 1.0	10.4 ± 1.0	9.5 ± 0.9	9.9 ± 1.0	12.7 ± 0.5	11.1 ± 0.4	11.9 ± 0.5
36–75	54.7 ± 2.9	43.9 ± 2.7	49.3 ± 2.8	49.3 ± 6.8	42.9 ± 3.3	46.1 ± 5.1	52.8 ± 4.2	41.1 ± 2.6	46.9 ± 3.4	50.1 ± 3.6	41.5 ± 3.0	45.8 ± 3.3	NA	NA	NA

Means of 5 pens±, no significant difference was observed. M: male; F: female; A: average. NA: not assessed.

**Table 5:** Productive parameters at slaughter time (75 days).

Parameter	px-NTG	NTG	AWPG	OF-AWPG
BWG	2697.75 ± 234.37	2448.10 ± 335.36	2671.10 ± 130.94	2611.04 ± 150.98
FC	2.66 ± 0.18	2.80 ± 0.36	2.51 ± 0.10	2.40 ± 0.12
IP	101.42	87.43	106.42	108.79

## Data Availability

Metagenomic data were submitted to the NCBI Bioproject under the Submission ID: SUB15225959 and the BioProject ID: PRJNA1245435.

## Supplementary Materials

The following supporting information can be downloaded at https://www.mdpi.com/article/10.3390/ani15121752/s1, Table S1: Main species identified in the organic fermented additive by metagenomics; Table S2: Villi Height (VH) and Crypt Depth (CD) at 6.5 and 19.5 dpi; and Table S3: Weekly productive parameters and productivity index.

## Abbreviations

The following abbreviations are used in this manuscript:

FPPS	Family poultry production systems
INTA	Instituto Nacional de Tecnología Agropecuaria
AE	Agroecology
LAB	Lactic acid bacteria
BWG	Body Weight Gain
rBWG	Relative Body Weight Gain
FC	Feed Conversion
LS	Lesion Score
PI	Productivity index
ACI	Anticoccidial Index
LDG	Low-dose group
IDG	Intermediate dose group
pHDG	pre-inoculated high-dose group
HDG	High dose group
VH	Villus height
CD	Crypt depth
AWP	Animal Welfare Practices
NTG	non-treated control group
AWP	animal welfare practice
AWPG	animal welfare practice group
OF-AWPG	AWP with organic fermented additive group
px-NTG	proximity non-challenged- non treated groups
NC	non-challenged-not treated group
