# Supplementary material for: Evaluating Good Husbandry Practices and Organic Fermented Additives for Coccidiosis Control in a Pilot Study Using Slow-Growing Broilers
Source: Animals (Basel). 2025 Jun 13;15(12):1752. doi: 10.3390/ani15121752 (PMC12189636; doi:10.3390/ani15121752)
Supplement: Supplementary file 1 [file animals-15-01752-s001.zip › animals-3651398-supplementary.pdf]

Table S1. Main species identified in the organic ferment by metagenomics.

Organism	Domain	Order	Abundance (%)
Lentilactobacillus buchneri	Prokaryote	Lactobacillales	62.04
Lacticaseibacillus paracasei	Prokaryote	Lactobacillales	11.93
Schleiferilactobacillus harbinensis	Prokaryote	Lactobacillales	3.31
Lactiplantibacillus plantarum	Prokaryote	Lactobacillales	1.14
Streptomyces cyaneogriseus	Prokaryote	Kitasatosporales	0.57
Zea mays	Eukaryote	Poales	2.42

Table S2. Villus Height (VH) and Crypt Depth (CD) at 6.5 and 19.5 dpi.

**6.5 dpi**	**VH (µm)**	**VH R (%)**	**CD (µm)**	**VH: CD**
NTG	927.79 ± 172.2^{a}	NC	NA	NA
AWPG	874.6 ± 199.9^{a}	NC	NA	NA
OF-AWPG	851.17 ± 223.8^{a}	NC	NA	NA
**19.5 dpi**				
NTG	871.88 ± 157.7^{a}	-6.03	317.96 ± 71.4^{a}	2.74
AWPG	989.61 ± 236.7^{b}	13.15	323.37 ± 84.4^{a}	3.06
OF-AWPG	964.19 ± 236.9^{b}	13.28	296.52 ± 72.09^{a}	3.25

Abr.: NC: not correspond; NA: not analyzed. VH: Villus height R: Villus height recovery; CD: crypt depth NTG: non-treated group; AWPG: animal welfare practices group; OF-AWPG: organic ferment-animal welfare practices groups. Results are expressed as the means ± standard deviation. Different superscript letters in each column and time point indicate significant differences ($p < 0.05$).

Table S3. Weekly productive parameters and productivity index.

Groups	BWG (g)	FC (g)	PI
**W01**			
NC	88.73 ± 4.68	0.98 ± 0.02	8.70
px-NTG	77.36 ± 1.37	1.25 ± 0.02	6.20
NTG	72.98 ± 4.75	1.21 ± 0.05	6.04
AWPG	70.89 ± 4.71	1.25 ± 0.05	5.66
OF-AWPG	65.79 ± 1.84	1.33 ± 0.07	4.94
**W02**			
NC	249.10 ± 14.72	1.33 ± 0.08	18.72
px-NTG	215.00 ± 8.35	1.43 ± 0.06	15.23
NTG	167.33 ± 13.18	1.63 ± 0.12	10.28
AWPG	178.84 ± 10.54	1.64 ± 0.06	10.92
OF-AWPG	164.67 ± 7.55	1.64 ± 0.13	10.02
**W03**			
NC	408.30 ± 23.68	1.83 ± 0.05	22.31
px-NTG	429.59 ± 29.22	1.72 ± 0.13	25.03
NTG	343.50 ± 18.45	1.78 ± 0.09	19.34
AWPG	381.56 ± 26.10	1.66 ± 0.07	22.98

OF-AWPG	364.21 ± 18.97	1.53 ± 0.02	23.80
**W04**			
NC	606.80 ± 55.18	2.16 ± 0.10	28.09
px-NTG	619.83 ± 38.22	1.89 ± 0.15	32.73
NTG	515.00 ± 29.70	1.90 ± 0.08	27.04
AWPG	535.69 ± 44.09	1.90 ± 0.16	28.20
OF-AWPG	520.54 ± 32.36	1.70 ± 0.07	30.70
**W05**			
NC	1260.20 ± 74.46	2.17 ± 0.11	57.95
px-NTG	1265.20 ± 74.46	2.18 ± 0.15	58.04
NTG	1098.68 ± 92.97	2.18 ± 0.15	50.37
AWPG	1133.64 ± 68.11	2.10 ± 0.08	54.07
OF-AWPG	1106.29 ± 75.31	1.85 ± 0.12	59.89
**W06**			
px-NTG	1265.20 ± 74.46	2.18 ± 0.15	58.07
NTG	1.098.68 ± 92.97	2.18 ± 0.15	50.37
AWPG	1133.64 ± 68.11	2.10 ± 0.08	54.07
OF-AWPG	1106.29 ± 75.31	1.85 ± 0.12	59.89
**W07**			
px-NTG	1537.20 ± 64.63	2.34 ± 0.18	65.74
NTG	1370.93 ± 136.14	2.34 ± 0.20	58.60
AWPG	1418.39 ± 85.64	2.28 ± 0.09	62.14
OF-AWPG	1390.79 ± 82.71	2.01 ± 0.15	69.17
**W08**			
px-NTG	1863.76 ± 113.85	2.62 ± 0.19	71.04
NTG	1695.10 ± 113.85	2.68 ± 0.22	63.16
AWPG	1725.14 ± 140.24	2.64 ± 0.25	65.31
OF-AWPG	1694.29 ± 95.37	2.46 ± 0.16	68.98
**W09**			
px-NTG	1944.38 ± 177.84	2.78 ± 0.22	69.87
NTG	1874.60 ± 184.80	2.81 ± 0.26	66.79
AWPG	1933.14 ± 110.27	2.66 ± 0.09	72.67
OF-AWPG	1920.29 ± 103.20	2.50 ± 0.15	76.87
**W10**			
px-NTG	2247.51 ± 131.64	2.76 ± 0.15	81.35
NTG	2100.52 ± 247.02	2.86 ± 0.30	73.51
AWPG	2159.14 ± 119.13	2.74 ± 0.08	78.77
OF-AWPG	2146.54 ± 122.96	2.57 ± 0.14	83.63
**W11**			
px-NTG	2697.75 ± 234.37	2.66 ± 0.18	101.42
NTG	2448.10 ± 335.36	2.80 ± 0.36	87.43
AWPG	2671.10 ± 130.94	2.51 ± 0.10	106.42
OF-AWPG	2611.04 ± 150.98	2.40 ± 0.12	108.79

Abr: BWG: body weight gain; FC: feed conversion; PI: productivity index; W: week, NC: negative control; px-NTG: proximity non-treated group; NTG: non-treated group; AWPG: animal welfare practices group; OF-AWPG: organic ferment-animal welfare practices groups. Results are expressed as the means ± standard deviation
